# A Model for the Detection of Moving Targets in Visual Clutter Inspired by Insect Physiology

**DOI:** 10.1371/journal.pone.0002784

**Published:** 2008-07-30

**Authors:** Steven D. Wiederman, Patrick A. Shoemaker, David C. O'Carroll

**Affiliations:** 1 Discipline of Physiology, School of Molecular and Biomedical Science, The University of Adelaide, Adelaide, Australia; 2 Tanner Research Inc., Monrovia, California, United States of America; Vrije Universiteit Amsterdam, Netherlands

## Abstract

We present a computational model for target discrimination based on intracellular recordings from neurons in the fly visual system. Determining how insects detect and track small moving features, often against cluttered moving backgrounds, is an intriguing challenge, both from a physiological and a computational perspective. Previous research has characterized higher-order neurons within the fly brain, known as ‘small target motion detectors’ (STMD), that respond robustly to moving features, even when the velocity of the target is matched to the background (i.e. with no relative motion cues). We recorded from intermediate-order neurons in the fly visual system that are well suited as a component along the target detection pathway. This full-wave rectifying, transient cell (RTC) reveals independent adaptation to luminance changes of opposite signs (suggesting separate ON and OFF channels) and fast adaptive temporal mechanisms, similar to other cell types previously described. From this physiological data we have created a numerical model for target discrimination. This model includes nonlinear filtering based on the fly optics, the photoreceptors, the 1^st^ order interneurons (Large Monopolar Cells), and the newly derived parameters for the RTC. We show that our RTC-based target detection model is well matched to properties described for the STMDs, such as contrast sensitivity, height tuning and velocity tuning. The model output shows that the spatiotemporal profile of small targets is sufficiently rare within natural scene imagery to allow our highly nonlinear ‘matched filter’ to successfully detect most targets from the background. Importantly, this model can explain this type of feature discrimination without the need for relative motion cues.

## Introduction

Certain flies (as well as other kinds of insects) detect and track small moving objects as they engage in rapid pursuits, demonstrating the capability to discriminate between targets (e.g. other flies) and an often cluttered, moving background [1], [2]. This is an especially challenging task considering that the fly compound eye limits visual resolution to ∼1° [3].

Neurons sensitive to (and in some cases selective for) small moving targets have been described in a variety of insect species [4]–[7]. Recent intracellular investigations have more carefully characterized a number of target-selective neurons in the optic ganglia of the hoverfly [8]–[10]. These ‘small target motion detectors’ (STMDs) were found to be exquisitely selective for small targets subtending no more than a few degrees of the visual field, equivalent to just one or two ‘pixels’ of the compound eye. The receptive fields of STMDs vary in size, with some extending just a few degrees, to those that encompass the whole eye hemifield. The target response may vary in magnitude across this region, however the size selectivity is independent of the target location [8] or the size and shape of the receptive field [9].

STMDs respond to targets moving relative to a background, in many cases when the background itself is moving [9]. Conceptually, it would seem likely that neural mechanisms required for such a task involve segregation of the motion of the target from the motion of the background. Surprisingly, whilst some STMDs exhibit a suppressed response in the presence of background motion, a subset respond robustly even when the targets move at the *same velocity* as the background, i.e. with no relative motion cues [9]. However, the response to wide-field background motion alone elicits no response. This implies that the spatial statistics of small targets, with respect to the background, form an important cue for discrimination, regardless of any additional role that may be played by other motion cues [9].

### Computational models for target discrimination

Understanding the computation that underlies small target selectivity and rejection of background motion presents a daunting challenge. Some models for target discrimination rely on inhibitory feedback of wide-field motion signals to localized motion detectors [11], [12], which may provide an explanation for small target selectivity, but would lead to inhibition by background motion. Another model, for what some thought at the time was the target selectivity of a higher order locust neuron [13], has lateral inhibitory interactions around a centre unit. This model was based on cells responding transiently to both contrast increments (ON channel) and contrast decrements (OFF channel) in a full-wave rectified manner. A lateral unit, derived from the local signal spread of these channels, was hypothesized to mediate the inhibitory interactions on these centre units [14]. Here we examine and model a similar neuron type we refer to as the ‘Rectifying Transient Cell’ (RTC). We show that fast temporal adaptation and lateral inhibitory connections, characteristic properties of RTCs, could provide the basis for an alternative model for small target selectivity, robust against wide-field background motion.

### Full-wave rectifying transient neurons

Extracellullar recordings in the first optic chiasm between 2^nd^ and 3^rd^ order interneurons of the fly brain (between the lamina and medulla), first showed the presence of “on-off” cells (Arnett fibers) with full-wave rectification [15], [16]. Surprisingly, these cells were later re-examined and shown to adapt independently to luminance changes, dependent on the polarity (increment or decrement) of the change [17]. This independent adaptation was also observed in medullary neurons in the locust [18]. These locust neurons had a ‘breakthrough response’ when stimulated with a pulse of the same polarity but greater contrast than the prior adaptor. The authors hypothesized that this nonlinear adaptation might enhance responses to the contrasting edges of visual features, whilst rejecting lower contrast “textual detail” [14], [19].

**Figure 1:**
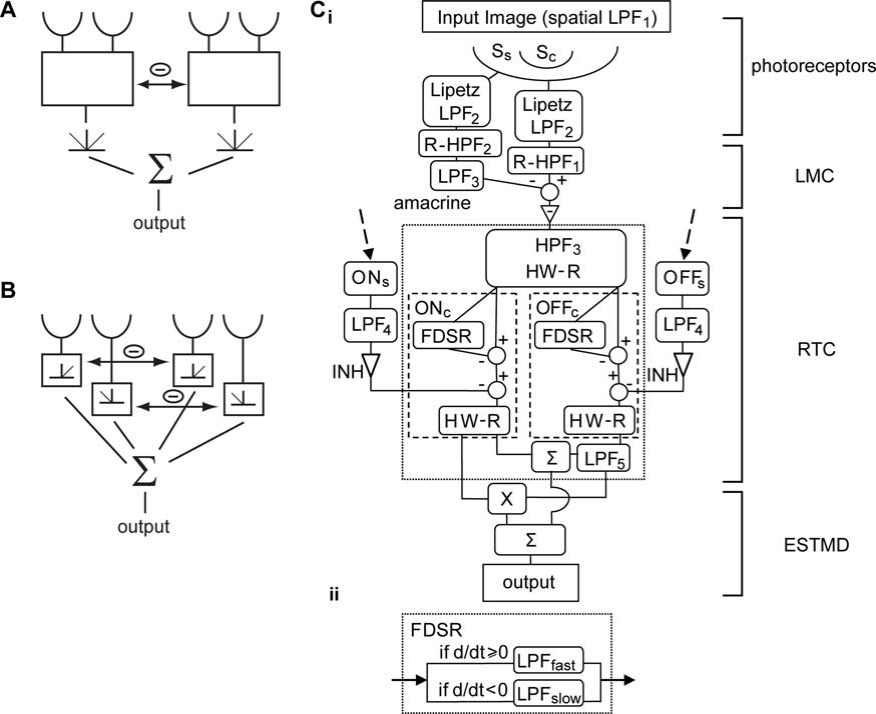
Model Overviews. (A) Model of the ‘on-off’ unit as described by Jansonius and van Hateren [21]. The transient subunits exhibit fast adaptation and lateral inhibition before full-wave rectification and spatial pooling. (B) Our proposed version of rectifying transient cells where fast adaptive units are segregated into ON and OFF channels via half-wave rectification. Each polarity channel laterally inhibits one another before spatial pooling. (Ci) The detailed block diagram of the elementary small target motion detector (ESTMD) model. Early visual processing (photoreceptors, Large Monopolar Cells (LMC) and amacrine cells) is modeled with optical blurring (LPF_1_), a nonlinear compressive transform (Lipetz function) with an adaptive mid-point parameter, and spatiotemporal band-pass filtering (LPF_2&3_, R-HPF_1&2_). The signal is separated into independent channels (responding to contrast increments and decrements respectively) via further high-pass filtering (HPF_3_) and half wave rectification (HW-R). Each channel exhibits fast adaptation, implemented via the FDSR inhibition (see Cii). The channels are separately inhibited by a delayed (LPF_4_) signal derived from surrounding channels of the same type. The strength of this surround inhibition is determined by the free gain parameter INH. To implement sensitivity to dark targets, the OFF channel is delayed (LPF_5_) and then recombined with an undelayed ON channel in either a linear (Σ) or quadratic (X) manner. (Cii) Fast depolarization, slow repolarization (FDSR). If the input signal is ‘depolarizing’ (positive temporal gradient), a first-order low pass filter with a small time constant (LPF_fast_) is used, otherwise for a ‘repolarizing’ signal (negative gradient) a larger time constant is applied (LPF_slow_). The resulting processed signal represents an ‘adaptation state’ which then subtractively inhibits the unaltered pass-through signal. [LPF_1_ Gaussian blur (half-width 1.4°); LPF_2_ τ = 2.5 ms; R-HPF_1&2&3_ τ = 40 ms, 30% DC; LPF_3&4_ τ = 2 ms; LPF_5_ τ = 25 ms; LPF_fast_ τ = 1 ms; LPF_slow_ τ = 100 ms; INH = 3 (free parameter)].

Spatial antagonism observed in the LMC, an earlier 1^st^ order interneuron in the lamina, appears to utilize inhibitory interactions between nearest neighbor receptors [20]. However in the ‘on-off’ cell experiments [21], separated pulses (5°) revealed antagonism on a larger spatial scale, equivalent to several facets of the compound eye. The authors proposed a model where rectification occurs after lateral inhibition of the subunits (Figure 1A), however, unless the inhibitory influence of neighbors is excessively strong, it is difficult to explain why the summing of spatially separated rectified signals, responding to pulses of ‘like’ sign, should produce an inhibition of the overall response as was observed [21]. These results lead us to propose the possibility of a second-order of local inhibitory interactions between ‘like’ ON channels and OFF channels, before they are recombined via spatial pooling (Figure 1B).

### Rectifying Transient Cells in the target detection pathway

We have developed a model for small target motion detection inspired by the properties of the higher order STMDs, and including a RTC-type component. We validate key stages of the model with intracellular recordings of the RTC in the fly (*Calliphora stygia*) medulla and with published physiological data. We investigate the temporal responsiveness of the RTC and obtain filtering parameters for the STMD model. We show that the properties of independent adaptation and lateral inhibitory interactions, as observed in ‘on-off’ cells and the RTC, are well suited for a role in target detection. We show that the spatiotemporal signature associated with the motion of a small feature is the passing of two contrast boundaries of opposite polarities (i.e. due to the leading and trailing edges), with limited spatial extent – which induces an excitatory response little affected by centre-surround inhibition or adaptation of the presumed ON and OFF channels. We include a stage for the recombination of ON and OFF channel signals, as yet untested by electrophysiological experiments, which enhances small target sensitivity. Finally, we show that this model leads to enhanced target discrimination, even when there are no relative motion cues between target and background.

## Methods

### Modeling

A model for an elementary small target motion detector (ESTMD) was created in Simulink (Mathworks), with image preparation and analysis tools programmed in Matlab (Mathworks). The term ‘elementary’ refers to a single unit that would be pooled to emulate the ‘position invariant’ nature of an STMD neuron [8]. Each major component in the model (Figure 1C) is inspired by key stages in visual processing and will be discussed in detail later.

We do not attempt to emulate biophysical properties of cellular dynamics, e.g. compartmental modeling, nor are we developing a neural network representation. Rather we are building a numerical model based on linear and nonlinear spatial and temporal filtering and typical feed-forward signal processing methods. This approach allows for the model to be implemented in engineering applications.

**Figure 2:**
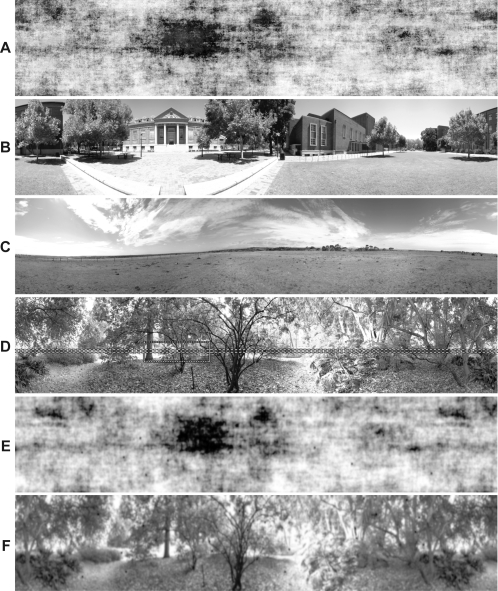
Input Images and Optical Blurring. Four panoramic images are used as model inputs to test target discrimination. The images display natural statistics with luminance intensity inversely proportional to spatial frequency [24]. Image (A) is composed of the average magnitude and phase of 13 natural images [22]. Image (B) includes several man-made structural elements. Image (C) is relatively sparse, whilst (D) is a highly cluttered scene. The images are panoramic and extend 72° vertically. They have a resolution of 2048×410 pixels, with the ‘green’ channel of the RGB image (depicted here in grayscale) retained for further processing, approximating the spectral sensitivity of motion detection mechanisms in the fly visual system[23]. The row section highlighted in image (D) corresponds to the data traces of Figure 9. Images (E) and (F) are the optically blurred versions of images (A) and (D), including 20 pseudo-randomly scattered targets (1.6°×1.6°) in each image.

**Figure 3:**
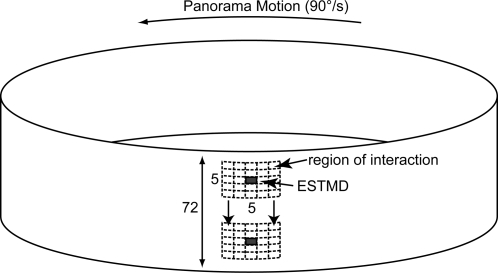
Panorama Rotation. A single model output has 25 ‘photoreceptors’ as inputs, each with 1° sampling separation (inter-ommatidial angle), thus represents a 5°×5° grid. The values representing luminance intensities at these locations vary over time as the panorama image is rotated past the ESTMD at 90°/s. Linear interpolation between pixels in the horizontal spatial domain results in higher temporal resolution (sampling at 5 kHz). There is an ESTMD at each degree separation down the vertical column, therefore 72 in all, each with overlapping, feed-forward, receptive fields.

The ESTMD model was tested using a series of panoramic images (Figure 2) (see Input Imagery) animated at a high temporal sample rate (5 kHz) to simulate continuous time. A 5×5 array of local ‘photoreceptor’ inputs was used to evaluate the response of the central ESTMD (Figure 3). Because the input imagery is a circular panorama, continuous motion allows estimation of the output of this ESTMD for all horizontal locations on the image. The region of interaction was shifted vertically in 1° increments to build up a 2 dimensional representation of ESTMD outputs in a raster fashion (Figure 3). The stimulus was rotated at 90°/s (within the optimal range for STMD neurons [8]) for two complete revolutions, with the first discarded, to avoid start-up transients.

### Input imagery

To test for robustness of the model for discriminating targets embedded in visual clutter, a series of three panoramic images (Figure 2B–D), with a 72° vertical extent, were acquired from natural habitats [22]. The 8-bit images were 2048×410 pixels. Although original panoramas were sampled as RGB, all simulations used the green channel only in order to approximate the spectral sensitivity of the fly photoreceptors that subserve motion processing [23]. A fourth image (Figure 2A) was obtained by combining the three natural images with ten others and averaging their phase and magnitude in the frequency domain [22]. This combined image, whilst displaying a typical power spectrum, lacks hard edge-like contours found in many (but not all) natural images. This image acts as a control with respect to potential phase congruency components underlying motion detection mechanisms. The first stage of modeling emulates fly optics via spatial blurring (see Results: Photoreceptors), therefore reducing hard edges, including those of the targets. The Gaussian blurring is shown for two images (Figure 2E, 2F) with 20 scattered 1.6°×1.6° targets embedded. The targets effectively have varying contrasts, and are difficult to discern, revealing the challenging nature of this target discrimination task. All four of the images had power spectra showing an approximately 1/*f* dependence on spatial frequency *f*, which is typical of natural images [24], [25].

We created a second set of images, identical to the first, but into which black targets (1.6°×1.6°) were pseudo-randomly distributed, with each target centered on an ommatidial row. To improve computational efficiency, we inserted twenty such targets into each image. We maintain a 70° horizontal separation between the targets and a 6° vertical separation. This limits spatiotemporal interactions between the targets at any stage of the modeling, with a larger (in effect longer) horizontal separation required, as this becomes the resultant temporal domain due to the panorama being rotated horizontally (influences are of longer-term adaptive components, e.g. photoreceptor dynamics). Because these targets become a feature of the image (i.e. there is no relative motion between targets and background) these simulations test the most demanding condition observed in physiological STMD experiments [9].

Model simulation was run with a single control trial using the original panoramas without targets and 26 trials (for each image) in which different pseudo-random target distributions were used, allowing us to evaluate responses from a total of 26×20 target locations, across four images.

To analyze how effectively targets are discriminated a spatial image of the model output, at varying stages of processing, was reconstructed from the vertical columnar units, and binning of the horizontal time dimension (back into an equivalent 1° spatial domain). Target locations are determined taking the non-uniform lag into account. We determine ‘hits’ (above threshold output corresponding to target location) and ‘false positives’ (above threshold outputs not corresponding to target locations). This categorization is done for each image (Figure 2), at each processing stage, and across varying model output thresholds. By varying this threshold and plotting ‘hit’ rate (relative to total targets present in the scene) versus number of ‘false positives’, we constructed Receiver Operating Characteristic (ROC) curves.

In addition to the experiments using natural images, basic characteristics of the ESTMD were evaluated using a similar stimulus paradigm into which targets of varying contrast, height and velocity were animated against bright or mean luminance backgrounds.

### Physiology

Flies (*Calliphora*) were either caught in the wild or reared in the laboratory under a natural day/night cycle. Insects were immobilized with wax. The back of the head was shaved, and a small hole in the cuticle was removed. Air sacs and other tissue were removed to provide clear access to the medulla. The brain was immersed with a Ringer solution: NaCl (130 mM), KCl (6 mM), MgCl_2_ (4 mM), CaCl_2_ (5 mM), with HEPES buffer at pH 7.0. Osmolarity was adjusted to 450 mM with the addition of sucrose. The fly was positioned to view a 200 Hz CRT monitor, mean luminance of 100 cd m^−2^. The visual stimuli were programmed in Python, using the VisionEgg stimulus software (www.visionegg.org).

Micropipettes were pulled from 1 mm (O.D.) thick walled alumina-silicate glass capillaries (SM100F-10, Harvard Apparatus Ltd.), on a Sutter Instruments P-97 puller, and filled with 2 M KCl or 2 M potassium acetate. Electrode resistances were typically 120–150 MΩ.

A wide-field, square-wave, flicker stimulus (1 Hz) induced opposing polarity potentials within the extracellular space. Intracellular recording from the RTC was identifiable by: a) a drop to resting membrane potential of approximately −60 mV; b) the full-wave rectification of the signal; c) depolarizing responses of 10–15 mV (graded), with ∼10 mV spikelets. The data were sampled at 5 kHz during acquisition, using a National Instruments 16-bit ADC. Data analysis was performed offline with Matlab.

## Results

We consider here in detail both the major stages of our model, and compare their outputs with known biological counterparts.

### Photoreceptors

After target insertion we low-pass filter input images (Gaussian, half-width 1.4°) to mimic the spatial blur of fly optics (Figure 1C, LPF_1_) [26]. Luminance values sampled by “photoreceptors” at 1° spatial separation approximately match the resolution of *Eristalis* [27] and *Calliphora* [3]. For computational efficiency, we use rectangular sampling in a 5°×5° receptor patch, rather than emulating the hexagonal distribution of ommatidia (Figure 3). Photoreceptor transduction transforms the input luminance to membrane potential in a roughly logarithmic manner around an operating point determined by stimulus history [20], [28], [29]. Our model mimics this effect by transforming luminance values with a Lipetz function (Equation 1), with the exponent *u* set at 0.7, as in our earlier modeling of fly motion detection [30].
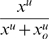
(1)


To elaborate this Lipetz nonlinearity we include an ‘adaptation state’ with the mid-level parameter x_0_ set as a first-order low-pass filtered version of x (time constant (τ) of 750 ms). Fly photoreceptor responses are temporally limited, with a corner frequency of 40–70 Hz [31]. To capture this, our modeling employs a static low-pass filter with corner frequency of 60 Hz (Figure 1C, LPF_2_) following the Lipetz transform.

### Large Monopolar Cells

While the role of LMCs in motion processing has been controversial, most research suggests that they are the ideal input to this pathway [32]–[34]. The LMCs have been shown to remove redundancy [20] and maximize information transmission [35] and they work as spatiotemporal contrast detectors, suitable for feature detection. Therefore, we implement an LMC-like spatiotemporal band-pass filtering on the photoreceptor output (Figure 1C). Spatial antagonism can be modeled as a recurrent inhibitory network (direct LMC to LMC inhibition), however, surround inhibition in a feed-forward manner, via a proposed surround ‘amacrine cell’ is equally plausible and is in accord with recent research on fly retina-lamina circuitry [36]. Our modeling comprises an amacrine cell that samples the surrounding nine photoreceptor outputs and subtractively inhibits the centre LMC (leaving a 30% DC spatial component). LMC spatial filtering dynamics are variable, dependent on overall light adaptation levels [37]; however, our model parameters remain constant for computational efficiency. The inhibitory signal is delayed prior to the subtraction by application of a first-order low-pass filter (LPF_3_, τ = 2 ms), representing the time course of the amacrine cell signal spread [38]. The LMC has band-pass temporal characteristics, with low frequency roll-off below a few hertz and high frequency at ∼80–100 Hz, in light adapted conditions [39]. For our model, the LMC signal is temporally filtered (R-HPF_1_) with a ‘relaxed’ first-order high-pass filter (one that passes a small DC component of 10%). This filter is characterized in the Laplace domain by the transfer function:
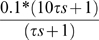
(2)where s is the Laplace variable and τ = 40 ms. The LMC signal is inverted, to replicate the hyperpolarizing response to luminance increments observed in intracellular recordings [40].

### Rectifying Transient Cell

**Figure 4:**
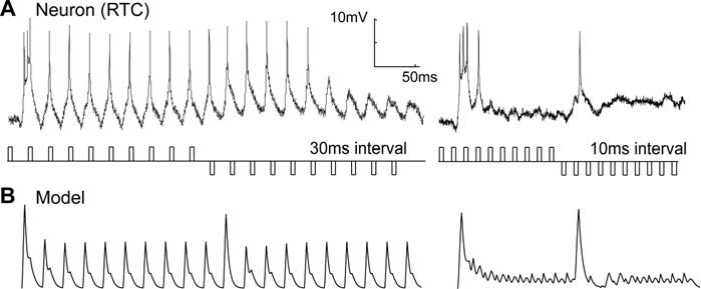
Rectifying Transient Cells (Independent Adaptation). (A) Intracellular recording from a rectifying transient cell shows independent adaptation to ‘on’ and ‘off’ contrast changes. Pulses of equal, but opposite, contrast polarity are of 5 ms duration and separated by varying intervals. At a pulse interval of 30 ms (left trace), little adaptation can be seen (but the response to the first pulse is strongest). At 10 ms interval, temporal adaptation limits the response *until the polarity of the contrast reverses*, which then produces an unadapted response. A single electrophysiological recording is shown for clarity (from N = 5). The model version (B) output is similar, capturing the important functional characteristics of the RTC.

Because electrophysiological data suggests that RTCs give little sustained response, unlike LMCs (Figure 4A), the signal is passed through an additional first-order high-pass filter (HPF_3_,τ = 40 ms). A half-wave rectification is performed to segregate ON and OFF channels of the input waveform, with the negative phase inverted in sign.

For each independent channel of the RTC, a signal representing an ‘adaptation state’ is formed by applying a nonlinear low-pass filter to the input signal with a fast onset, slow decay characteristic (Equation 3).
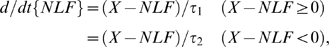
(3)X designates the input, NLF the filter output, and τ_1_ is set to 1 ms (LPF_fast_) and τ_2_ to 100 ms (LPF_slow_). Such a filter is an approximation to plausible biophysical mechanisms, such as an interneuron with a long intrinsic membrane time constant and strong, ‘bursty’ inputs. This fast depolarizing, slow repolarizing signal is subtracted from the unaltered, pass-through version of the input signal.

In addition to this step, a second subtractive inhibition is applied based on the average of the surrounding input signals of the same channel polarity (surrounding ON subtractively inhibit the centre ON channel and similarly for the OFF channels). This is based on the surround inhibitory effect found in the ‘on-off’ cells [21]. Unlike the previous parameters in our model, we do not have a physiologically derived estimate for the strength of this inhibitory effect, and consider the scaling of the inhibitory signal a free parameter (INH) in our modeling and simulations. Alteration of this value can be used to tune the model to different size image features. We include a neural delay, modeled by a first-order low-pass filter (LPF_4_, τ = 2 ms), which is applied to the averaged and scaled surround inhibitory pathways.

The channels are then half-wave rectified to mimic a thresholded response (a nonlinearity seen in many spiking neurons). The resultant channel signals are passed through a ‘neural delay’ smoothing filter (τ = 2 ms). This smoothing better represents the temporal response dynamics seen in the physiological RTC.

The final stage of processing is a recombination of the ON and OFF channels to form a single output corresponding to the ESTMD response. The simplest operation to achieve this would be a straightforward sum of the two output signals. However, we consider an operation that enhances selectivity for small, dark targets. A delay operator D[*], consisting of a low-pass filter (LPF_5_), is applied to the OFF channel prior to recombination with the undelayed ON channel. For generality, we took a phenomenological approach to this recombination allowing second-order as well as linear interactions:
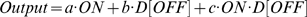
(4)


In our simulations, we consider primarily the purely linear case (*c* = 0), which we refer to as ‘RTC’, and the second-order case (*a* = *b* = 0), referred to as ‘ESTMD’. Note the formal similarity of the second-order structure to the correlational or Hassenstein-Reichardt elementary motion detector [41]. However, in this case the correlation operates on rectified signals of opposite polarity from the same spatial location, rather than signals from spatially neighboring locations. In this form, although tuned primarily to small contrasting features, this rectification of polarities resembles models proposed to explain selectivity for expanding edges in ‘looming’ motion detectors such as in the locust LGMD/DCMD [42], [43].

Although STMDs respond better to black targets [9] and light target sensitivity is not modeled here, a symmetric correlation operation could be established for a white target detector by interchange of the signal roles in Equation 4. This would provide white target sensitivity by correlating a delayed ON channel with an undelayed OFF channel. A detection mechanism for targets of both contrast polarities (light and dark) would involve summing these two versions or having any weighted combination of the above terms (both linear and second-order).

### Comparison of model responses to fly RTCs

We compare our recordings of the RTC in the medulla of the blowfly to our modeled responses. The intracellular recordings (Figure 4A) show independent adaptation to contrast increments and decrements, as seen in ‘on-off’ type cells [17], [19]. Figure 4A shows an experiment with a train of contrast pulses at two different frequencies. At 30 ms separation, the neuron recovers to produce graded depolarization in response to each pulse. When the separation is reduced to 10 ms, the adaptation suppresses the response to the stimulus. However, when the contrast polarity is reversed (from contrast increments to decrements), an unadapted response is observed before the neuron again rapidly adapts to the new polarity stimulus.

### Temporal responsiveness

Although our model captures the basic behavior of the biological RTC, the incorporation of an LMC-like input stage is somewhat contradicted by earlier work suggesting the frequency response (to sinusoidal stimuli) of ‘on-off’ units rolls off above 12 Hz [17], while the LMCs have a much higher corner frequency [39]. Jansonius and van Hateren [17] suggested this apparent low-pass characteristic is simply a result of the rapid adaptation that occurs at higher stimulus frequencies (as seen in Figure 4A); it is possible that the unadapted system has a much higher temporal acuity than this result would suggest.

**Figure 5:**
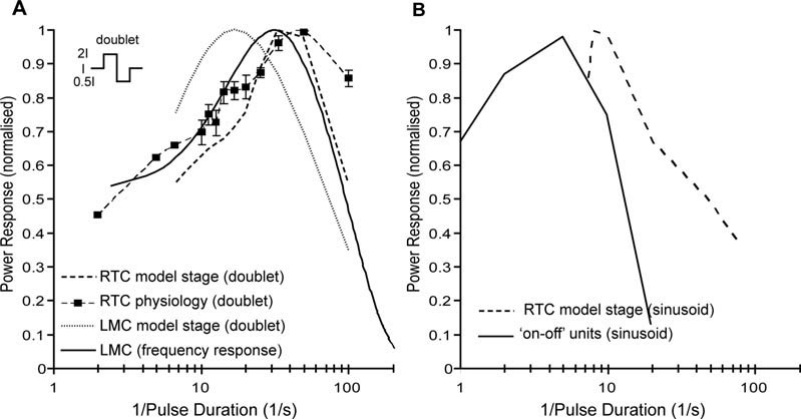
Temporal Responsiveness. (A) The response of the physiological RTC to a stimulus doublet (2, 5, 6.6 Hz N = 2, no error bars; others N = 6 (flies) mean±SEM). This RTC transfer function peaks at ∼50 Hz (dashed line, squares) and is still responsive at the highest stimulus frequencies, which are limited by the CRT refresh rate. We simulate doublet input and show that the model RTC frequency response is comparable to the physiological correlate (dashed line). The response to the doublet at the LMC stage of the model is also shown (dotted line). Frequency response properties of fly LMCs obtained via white noise analysis [38] is also plotted for comparison (solid line). It should be noted that the RTC and LMC response characteristics show a similar temporal responsiveness. (B) Previous analysis of ‘on-off’ units in the fly lamina [17] showed poor temporal responsiveness (peak at ∼6 Hz) (reproduced here, solid line) and our model shows a similar shift in response to the non-optimal sinusoidal stimulus (dashed line).

To test this hypothesis we used a ‘doublet’ stimulus consisting of a pair of pulses (‘on’ followed by ‘off’). Whilst not strictly containing energy at a single frequency, this stimulus allowed us to construct transfer functions for the RTC to a single stimulus cycle, thus avoiding the influence of adaptation. The response power is calculated as the mean-square value until the neuron returns to within 5% of the resting membrane potential. As can be seen in our physiological data (Figure 5A, dashed line, squares), the response of the medulla RTC to the doublet stimulus has a peak at high frequency (∼50 Hz). The RTC still responds with 85% maximum at 100 Hz, the highest frequency doublet that we could generate on our 200 Hz stimulus display. The model RTC (Figure 5A, dashed line) gives a similar temporal responsiveness. The RTC frequency response is a good match for that obtained by Fourier transforming the linear kernel for fly LMCs using white noise stimuli (Figure 5A, solid line) [38]. Interestingly, if we simulate the earlier experiments of Jansonius and van Hateren [17] with a wide-field sinusoidal stimulus (Figure 5B, dashed line), we obtain a curve that rolls off at a much lower frequency, consistent with their experimental data (reproduced in Figure 5B, solid line). Our model rolls off more sharply at low frequency, likely due to the pure nature of our high-pass filter (HPF_3_) and because the non-linearity introduced into their extracellular recordings by the thresholding mechanism for spike generation may lead to overestimation of weak responses. We conclude that the apparent low-pass nature of the ‘on-off’ cell frequency response was, as hypothesized, a result of adaptation [17], and that the response to transient as opposed to stationary stimuli reflects a much more rapid temporal response capability. Also, the inclusion of an LMC-like input stage in our model is supported by the very similar temporal characteristics of the LMC to the fly RTC.

### Contrast Sensitivity Function

The high-pass nature of the RTC data (and as captured by our model) we expect to form an ideal basis for a neural pathway for small target detection as the signal from the passing target boundaries provides a near optimal transient stimulus, with no spatial antagonistic suppression that would occur with larger features.

**Figure 6:**
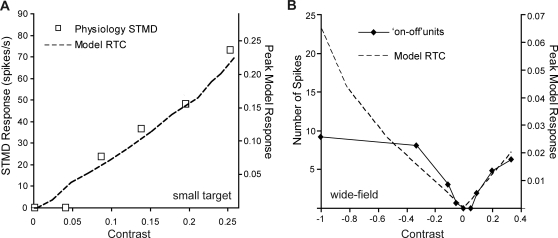
Contrast Sensitivity Function (Small Target and Wide-field). (A) The contrast sensitivity function is calculated from the peak model responses to varying contrasts of small targets (0.8°×0.8°) moving at 50°/s on a mean background (RGB 0.5) (dashed line). Also plotted (squares) are physiological STMD responses to a 0.8°×0.8° target, moving at 50°/s. However, this stimuli included a complex moving background (mean luminance 150 cd m^−2^, 45°/s) [9]. For the model we measure contrast as the effective neural contrast. For the physiological data the contrast values represent average Michelson contrasts as the targets transverse a complex moving background [9]. (B) Reproduced responses of ‘on-off’ units to wide-field contrast steps of 500 ms duration (solid line) [17]. For comparison, we plot the model RTC responses to a simulation of this wide-field visual input (dashed line). We note that low contrast sensitivity is observed in the model output due to spatial antagonistic interactions and this could be a plausible explanation for low contrast sensitivity in the ‘on-off’ units. The model responses in (B) are less than 1/10 those seen in response to small targets of equivalent contrast (A).

We determine the model response to a small target (0.8°×0.8°) as a function of target contrast and compare it with that induced by wide-field flicker stimuli (Figure 6). As the target is below the size of a single ommatidium, an effective neural contrast is calculated by the convolution of the target with the optical blurring filter (half-width 1.4°) [9]. Even very low contrast discrete targets induce a model response over 10 times higher than that of the wide-field flicker stimulus (compare at equivalent contrast Figure 6A with 6B, dashed lines). We also plot reproduced STMD responses to targets of varying contrast (Figure 6A, squares). However, it is important to note that these responses were to 0.8°×0.8° targets (50°/s) moving on complex moving backgrounds (45°/s) [9].

Physiological data for the low contrast sensitivity of ‘on-off’ cell responses to wide-field flicker [21] is well explained by the model (Figure 6B). The divergence seen between the model and neuron recordings at higher negative contrast is expected, since we make no attempt to account for saturating nonlinearities in neural components that would be expected in the biological system. Interestingly, the RTC model stage also produces a reasonable explanation for the near threshold contrast sensitivity of higher order STMD neurons (Figure 6A, squares) [9].

### Target height tuning

A feature of our ESTMD model is the inclusion of second-order spatial (lateral) inhibition by neighboring RTCs and a temporal cross correlation of the outputs of local ON and OFF pathways which form a ‘matched filter’ for both the spatial and temporal characteristics of small, moving features.

**Figure 7:**
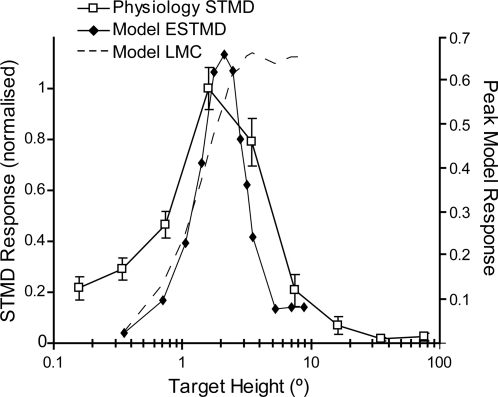
Target Height Tuning. The curve shows model ESTMD responses to targets of varying height (0.8° wide black targets on white background, moving at 50°/s). Physiological data from STMD neurons in the hoverfly for the equivalent target parameters and background is reproduced [10]. The model is selective for targets of less than a few degrees height with the suppression to the right of the peak determined by the strength of the lateral inhibition between channels. The model response to targets at the LMC stage (no units) shows that the responses remain at maximum as the target is extended vertically (max height shown of 10°), i.e. the LMC is not target selective. This highlights that a second-order spatial antagonism is required for target selectivity.

By analogy to models for direction-selective motion detectors where wide-field optic flow can be deduced by summing output of local elementary motion detectors, we use the term ‘elementary small target motion detector’ (ESTMD) for this stage. Responses of higher-order STMDs should be easily explained by simply summing across a weighted array of such ESTMDs to produce receptive fields of varying size (as observed in electrophysiological recordings from the lobula) [9], [10] whilst retaining position invariant selectivity for small features [8]. To confirm whether our model displays size selectivity, we estimate responses to discrete moving targets of different length (i.e. extended orthogonal to the direction of motion). Figure 7 shows that the ESTMD stage of our model provides an excellent fit to the data published for lobula STMD neurons [10]. Note that while LMCs act to maximize information to the higher order pathways by enhancing edge-like features, the very sharp suppression of responses to targets above a few degrees in size that characterizes both model and neuron responses cannot be explained by the simpler spatial antagonism of LMCs (Figure 7, dashed line).

### Velocity tuning

**Figure 8:**
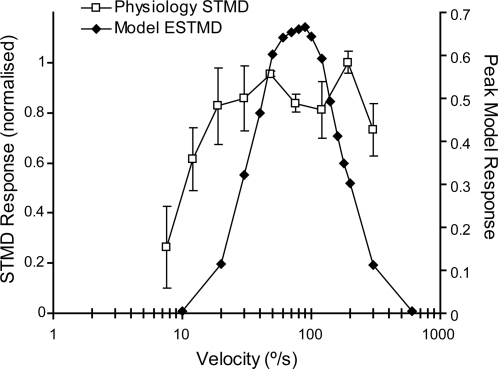
Velocity Tuning. Model ESTMD responses to black moving targets (0.8°×0.8°) on a white background at varied velocities is shown in comparison to the physiological data of STMD neurons to the same visual stimulus from hoverflies [8]. The model output exhibits typical velocity tuning as observed in correlation-type motion detection mechanisms. The tuning of the model parameters (particularly, the OFF delay filter time constant) determines at which point the velocity response peaks. The broadness of the tuning curve may be extended and shaped via the addition of a final saturating nonlinearity, not included in this model.

An important aspect of the second-order configuration of our model is its inherent similarity to a Reichardt correlator [41] such that the velocity dependence in response to small moving targets is essentially the same. The responses to a 0.8°×0.8° moving target (Figure 8) represents a typical velocity tuning curve as obtained from a delay-and-correlate-type model. The position of the peak response is dependent on the time constant of the delay filter D[*] (LPF_5_). For comparison, we plot the velocity tuning curve seen in STMD neurons [8]. We have not attempted to specifically fit this data (nor in the target height tuning) and note that differences in the broadness of the tuning curves could reflect additional compressive nonlinearities which we have not attempted to account for in this model.

Although the ESTMD model provides a good account for the basic tuning properties of STMDs, it is not unique in this respect. Other STMD models [11], [12] should also be able to explain both contrast sensitivity and velocity tuning. However, our key finding is that the unique adaptive component of the RTC inputs to our STMD can also explain the otherwise enigmatic finding that STMDs can respond to features embedded in clutter, but without *relative motion* cues [9].

### Responses to targets in clutter

**Figure 9:**
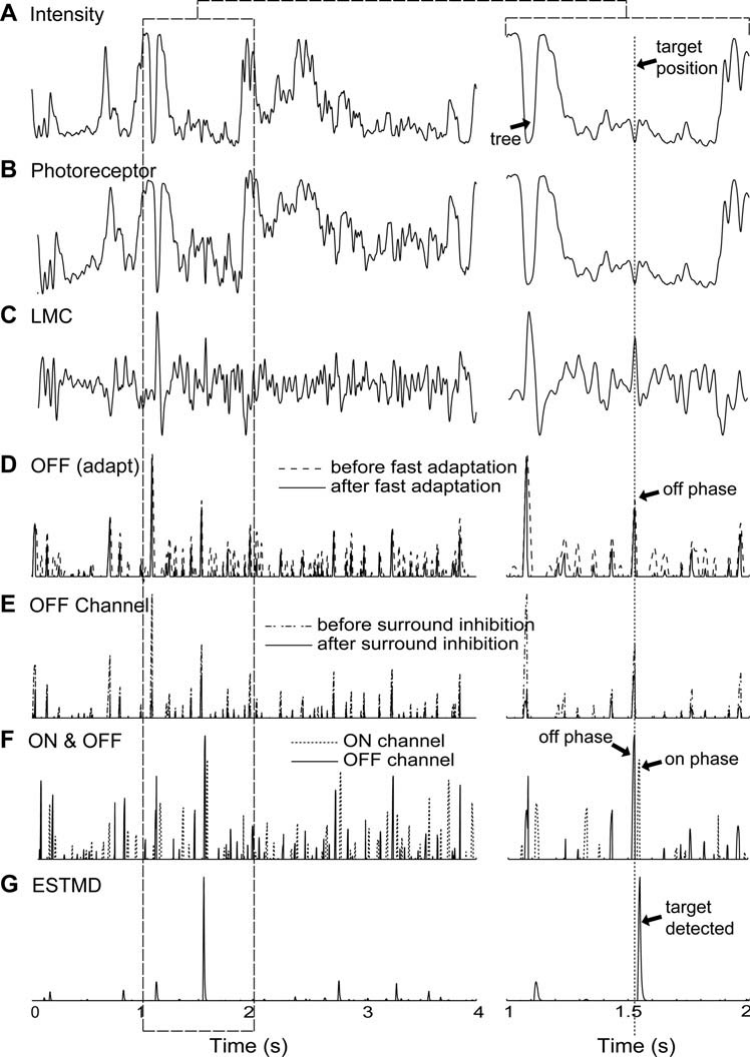
Sample Data Traces. The traces show the mode outputs at various stages of processing over the second complete revolution of the scene (at 90°/s). The right hand trace shows a magnified version of the period between 1 and 2 seconds. The y-axes are unit-less model outputs. The input intensity (A) is delineated in Figure 2D. Photoreceptor dynamics (B) encode a large luminance range into the limited dynamic range of the neuron. The LMC (C) exhibits spatiotemporal high-pass filtering, enhancing contrast boundaries. The temporal adaptive mechanisms within the independent channels suppress rapid texture variations but signal a novel contrast change (D). Surround antagonistic interactions limit the spatial extent of this type of signaling (E) and a final linear or quadratic recombination of the channels (F) signals the presence of a dark moving target (G).

Figure 9 shows a single output row at each stage of the model, in response to a panoramic image in which a small target is inserted (the image row is delineated in Figure 2D). We selected this row to illustrate the effect of the key stages of the model in enhancing target responses, whilst rejecting other high contrast features.

Photoreceptor dynamics encode a large luminance range into the limited dynamic range of the neuron [29], [44]. Our inputs have already emulated a similar process via a form of global gain control inherent in digital camera processing (Figure 9A–B). The LMC output (Figure 9C) with spatiotemporal high-pass filtering, enhances contrast boundaries in both space and time. The OFF fast temporal adaptation (Figure 9D, solid line) suppresses textures and signals larger ‘breakthrough’ contrast changes. The surround inhibition ensures that this effect is spatially localized. Note that the response to the tree trunk (t = 1.1 s), which also has a novel ‘off’ shortly followed by an ‘on’ contrast boundary is suppressed as a consequence of second-order spatial inhibition (Figure 9E, solid line). The ON channels (not shown) show similar characteristics. Finally, the OFF channel (Figure 9F, solid line) is temporally delayed and correlated with the undelayed ON channel (Figure 9F, dashed line) to signal target-like events (Figure 9G).

**Figure 10:**
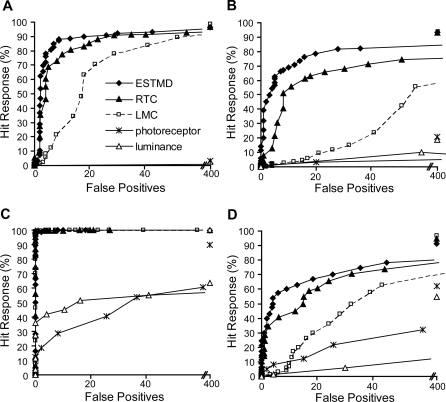
Natural Image Panoramas. Twenty targets (1.6°×1.6°) pseudo-randomly scattered (26 trials) on four panoramic images with the simulation run at a velocity of 90°/s. (A) Receiver Operating Characteristic (ROC) curves are shown for a scene with averaged natural statistics. The addition of RTC-type processing (solid line, triangle) to the LMC (dashed line, square) shifts the ROC curve to the upper left, revealing enhanced target discrimination. The quadratic (ESTMD) version of the model (solid line, diamond) shows further improvement via the multiplicative interaction of the delayed OFF and undelayed ON channels. (B) The LMC stage has a large number of false positives, due to high contrast, man-made features (which the RTC-type processing can discriminate). (C) This image is sparse so the targets can be readily discriminated by the LMC processing alone. (D) A highly textured scene, with many scattered targets losing defining characteristics, however, the target discrimination is still improved. Error bars are within symbol representation, therefore removed for clarity.

Figure 10 shows ROCs for the four panoramic images, at a velocity of 90°/s. During the pseudo-random distribution, some targets are scattered onto backgrounds of the same luminance (as the target) such that that they lose all defining characteristics. In image D (Figure 2), the most highly textured scene, it is difficult for the human observer to detect the scattered targets. Image C is extremely sparse and LMC filtering is enough for successful target discrimination (Figure 10C). Across the varied scenes, both linear (RTC) and quadratic (STMD) processing have improved the discrimination of targets as revealed by the shift to the upper left corner of the ROC curve (Figure 10 A–D). The limited number of false positives in the final model output suggests that target-like structures are rare in these natural image scenes.

These results show that a highly nonlinear filter (derived from the plausible biological components) exploits the spatiotemporal statistics of the moving target within its immediate surround. The statistics required are as follows 1) a small duration of time (∼50 ms) in which contrast changes do not exceed that of the upcoming target, therefore providing an unadapted ‘off’ phase. This provides ‘distinctiveness’ to the start of the dark feature. 2) An unadapted ‘on’ phase, which is inherent in the non-changing texture of the dark target. 3) These same characteristics, i.e. unadapted, opposite polarity, contrast changes, *to not be present* in the immediate surround. If this third characteristic were relaxed, the detector would be sensitive to a similar width/velocity profile as the target, though not suppressed by the height of the feature, i.e. the detector would also be stimulated by a vertical ‘bar’ stimulus.

### Relative motion

**Figure 11:**
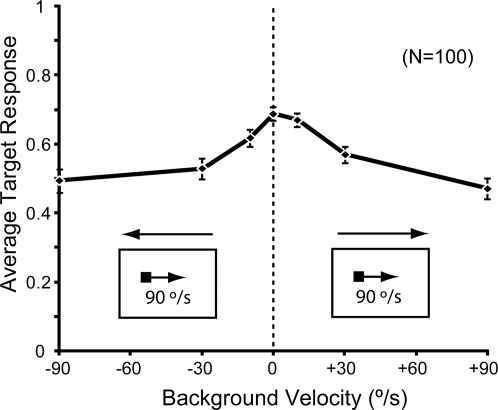
Relative Motion. Results of simulations carried out for 25 targets in each of the 4 images, where background speed and direction was varied. Target speed was constant at 90°/s (left to right, as indicated by pictograms). Data are mean±SEM, N = 100.

Intuitively, the ESTMD model is responsive to the motion of the contrast boundaries across the detector inputs. Relative motion between target and background will have an effect on ESTMD responses, as it alters the temporal statistics (dependent on background velocity) that establish the adaptation states of the independent channels. We tested this by varying the background motion with a constant target velocity of 90°/s (Figure 11).

Depending on background speed, we varied initial background position so that we could analyze target response at the same spatial juxtaposition of target and background (target size of 1.6°×1.6°). Hence, data for a background speed of +90°/s effectively represents the scenario in the other ‘no relative motion’ panorama simulations. We repeated this test in 100 distributed locations across the four panoramic images. We show that the ESTMD target responses are robust across the tested range of relative motions and the results confirm that the response improves when there is some relative motion, reaching a peak when the background speed is close to zero.

## Discussion

By recording from a cell in the fly medulla (the RTC) we have been able to determine their quick temporal responsiveness to transient stimuli. This has provided parameters, such as adaptation time constants, that form the basis of our target detection model. We have compared responses of the model, e.g. contrast sensitivity, height tuning, and velocity tuning, to those observed in STMD physiological recordings and find that they are well matched.

We have shown that linear systems analysis of steady-state responses is not an appropriate method for characterization of the relevant neural responses (Figure 5). In this case, when we consider the quickly adapting transient component of the RTC signal, we find the temporal responsiveness is well matched to presumed early components of the visual system (the LMC). We also observe that apparent contrast insensitivity of a neural system may be the result of wide-field antagonistic interaction. In our modeling, due to a neural delay in the surround interactions, the naturally ‘unrealistic’ wide-field flicker can transiently pass through the system, however with low contrast sensitivity the result. In comparison, the response to the limited spatial extent of a small target is very contrast sensitive.

Larger wide-field STMD neurons display a position invariant receptive field that typically spans many ommatidia [8]. This presumably requires a pooling of the outputs of many subunits from earlier stages of processing. In fact, because some STMD neurons are weakly direction-selective [8]–[10], rather than a summative pooling of ‘non-directional’ subunits, some type of higher-order spatial facilitation may take place. Weak direction selectivity could be built into our modeling via asymmetry in the inhibitory surrounds or via a higher order spatial facilitation during this pooling stage.

Unfortunately, RTC neurons are small and intracellular recording times are limited in duration. We have, to date, not been able to establish the morphology of the neuron via dye-filling techniques nor can we examine more time intensive spatial characterization stimuli. Nevertheless, we are confident from our dissection technique and precise control of the location of the pipette that our regular recordings from the RTC are from the medulla. However, we cannot be certain if they are intrinsic to the medulla, or if they reside elsewhere and project to, from, or via the medulla. There is the possibility that they may be the termination of the fibers identified by Arnett [15], [16] or a later postsynaptic element that has inherited the properties as seen in the projections from the lamina. Although our biological investigation of the RTC is limited, the aspects of computation that form the basis for our small target modeling has been well established in the work of Jansonius and van Hateren [17], Osorio [19] and now again in this present research.

### Conclusion

Our approach to modeling has provided a solution to the initially perplexing issue of how the STMD neuron responds robustly to target motion, even when there is no relative motion cue of the target to the background [9]. We have seen that this problem is solved by incorporating properties of the rectifying transient cell in the target detection pathway. This is an attractive solution, as our highly nonlinear matched filter is computationally less intensive than complex segregation of transparent motion fields, required for relative motion calculations.
